# 

**Published:** 1893-02-25

**Authors:** 


					February 25th
WITH EXTRA NURSING SUPPLEMEN
Per Annum, 8s. 6d., post free,
Monthly Parts, 6d.; post free, 8d.
the General Poet Office ai a Newspaper t>t Ditto per Aimunii 8s., post free,
"*nii3?ion in the United Kingdom and Abroad,]
ii0 Price twopence. TUfT''r?
& Vol. XIII.?Feb. 25th, 1893. rl JpM, ' M
{3^. at the General Port Office ai a Newspaper f it *? '
A
No. 335. Vol. XIII.J Saturday,
February 25th, It93.
[Twopence.

				

